# Closing the gap between formats for storing layout information in systems biology

**DOI:** 10.1093/bib/bbz067

**Published:** 2019-07-05

**Authors:** David Hoksza, Piotr Gawron, Marek Ostaszewski, Jan Hasenauer, Reinhard Schneider

**Affiliations:** 1 Luxembourg Centre for Systems Biomedicine (LCSB), University of Luxembourg, 6, avenue du Swing L-4367 Belvaux, Luxembourg; 2 Faculty of Mathematics and Physics, Charles University, Malostranské nám. 25, 118 00 Prague, Czech Republic; 3 Institute of Computational Biology, Helmholtz Zentrum München - German Research Center for Environmental Health, Ingolstädter Landstr. 1, 85764, Neuherberg, Germany; 4 Department of Mathematics, Technische Universität München, München, Germany; 5 Faculty of Mathematics and Natural Sciences, University of Bonn, Bonn, Germany

**Keywords:** network layout, molecular network, data format, systems biology, conversion

## Abstract

The understanding of complex biological networks often relies on both a dedicated layout and a topology. Currently, there are three major competing layout-aware systems biology formats, but there are no software tools or software libraries supporting all of them. This complicates the management of molecular network layouts and hinders their reuse and extension. In this paper, we present a high-level overview of the layout formats in systems biology, focusing on their commonalities and differences, review their support in existing software tools, libraries and repositories and finally introduce a new conversion module within the MINERVA platform. The module is available via a REST API and offers, besides the ability to convert between layout-aware systems biology formats, the possibility to export layouts into several graphical formats. The module enables conversion of very large networks with thousands of elements, such as disease maps or metabolic reconstructions, rendering it widely applicable in systems biology.

## Introduction

Systems biology aims at a detailed understanding of mechanisms underlying complex biological processes. To this end, systems biology combines the vertical approach, when a system is studied on different levels of detail, with the horizontal approach, when different aspects of a system at a given level of complexity are investigated. This combination of vertical and horizontal approaches often reveals complex mechanisms driving the behavior of a biological system. Such a complex system is then analyzed by computational methods and mathematical modeling approaches to propose an informed hypothesis about its functioning, to be subsequently validated in the wet lab [1]. A multitude of software tools focus on different facets of the field, creating a demand for standardization and interoperability efforts to enable seamless exchange of information about the underlying molecular systems. Visualization plays an important role in understanding of such systems, including graphic display of the parameters and results of simulations or the topology and layout of network models.

Layout is an important element for the analysis of complex networks. Proper visualization, including positioning of molecular entities, can greatly facilitate understanding of the entire system. Good examples are disease networks [2] or metabolic reconstructions [3], which can easily contain hundreds or thousands of entities. Layouts for these networks are usually manually encoded by a curator [4]. If this is not possible, one can rely on automatic layout to visualize a network. However, automatic layout has many issues, which still remain a challenge for the state-of-the-art algorithms [5]. Finally, the curator might prefer a specific layout to emphasize some properties or pathways of the system. For all these reasons, in the case of more complex systems, automatic layout is not possible and the network structure should be stored in a layout-aware format, i.e. a format allowing one to encode positions of molecular entities.

The most frequently used community-driven formats to encode molecular networks are the Systems Biology Markup Language (SBML) [6] and the Biological Pathway Exchange format (BioPAX) [7]. BioPAX is a knowledge exchange format used in pathway repositories such as Reactome [8] or Pathway Commons [9]. It allows the user to store the structure of a network, but not layout information. Therefore, it is not discussed here in detail.

SBML allows for storing both the structure of a network and its layout.

SBML is an XML-based format, and the SBML core provides a set of basic XML elements to encode and annotate molecular entities and their interactions. The core is complemented with extension packages that allow for specification of additional properties, such as quantitative or visual aspects of the networks or more complex entity-related properties such as residue modification or molecular complexes [10]. Specifications of individual extensions are independent of the core and thus add extension-specific information to existing molecular representation. These extensions do not affect tools that do not support them. One such SBML extension is the layout package (see Encoding systems biology layouts for a more detailed description), which allows for encoding positions of molecular entities and their interactions.

The ability to extend the SBML core with packages was introduced into the standard with SBML Level 3 (announced in 2010 [10]) with the layout package published in 2013 [11]. The package provides format for encoding positions of species, reactions, labels and compartments, but it does not specify how to encode visual properties of those entities, such as shape, color or thickness of edges. This became possible with the render package, an adjunct to the layout package, which was introduced in 2017 [12].

Due to the relatively late adoption of the layout and render packages, other formats filled the gap. Specifically, in 2009 the Systems Biology Graphical Notation (SBGN) was released [13]. Unlike SBML, SBGN does not describe a data format, i.e. a concrete encoding of element of molecular networks, but focuses on standardization of how networks are visualized. The language specifies a set of symbols for displaying molecular entities and processes. The difference between SBML render package and SBGN is that the former specifies encoding for shapes and other visual aspects of the elements, but does not regulate their appearance in any manner. The latter defines how entities and processes should look like, but does impose any encoding. In order to standardize the encoding in SBGN, an XML implementation of SBGN language, called SBGN Markup Language (SBGN-ML), was introduced in 2012 [14].

Even though SBGN-ML became available 3 years before the SBML layout package, it was not the earliest SBGN-inspired network layout solution. Seven years earlier, in 2005, CellDesigner [15], a very useful diagram editor for designing systems biology molecular networks, was introduced. CellDesigner features its own format, which is a software-specific extension of SBML, consisting of CellDesigner-specific XML tags within the SBML document. Because the last version of CellDesigner was released in 2014, it only supports (export to) the SBML Level 3 core format without layout. The layout information is stored in the CellDesigner’s SBML extension. Due to CellDesigner’s ubiquity, its particular format is accepted by many existing tools that are thus able to parse a layout encoded in CellDesigner files.

In consequence of this historical development, there are three closely related formats for capturing layout information: SBML layout and render extension, CellDesigner’s SBML extension and SBGN-ML. In this article, we present a review of these layout-encoding notations. We describe general characteristics of these notations (Encoding systems biology layouts) and overview their implementation in existing software tools and repositories (Support of layout in existing tools and repositories). We argue that, to the best of our knowledge, none of the existing visualization and editing tools supports full range of the formats within its import and export routines. To address this situation, we introduce a tool, which can be used to convert between these formats (Conversion module). This new tool is available via a REST API of a platform for visualization of molecular interaction networks [16] and allows for conversion between three layout formats. Next, we discuss the current state of layout encoding formats (Discussion) and conclude the article (Conclusion).

## Encoding systems biology layouts

A format capable of storing a diagram layout needs to handle not only information about the network structure but also positions of elements and labels, space they occupy and how they should be rendered, i.e. their shape, color and other visual aspects. Exchange formats therefore need to be able to encode the network structure together with the position and rendering information for network elements and interactions.

Unlike traditional graphs, where nodes can be simply encoded as points in space connected by simple lines, graphs in systems biology are more complex, featuring compound objects and their relations, which in turn requires a more complex language to describe them.

Systems biology networks following the SBGN guidelines comprise three types of entities (nodes): species, complexes and compartments. By species, we understand objects on molecular or cellular scale, like genes, proteins, RNAs or simple molecules. Furthermore, we consider phenotype as objects. These are nodes, representing an entire sub-process in the diagram. In general, species are simple objects, which can be positioned in space and their type can be visually encoded by a specific glyph (marker) or by any visual attribute such as color or fill pattern. A complex on the other hand has internal, potentially hierarchical, structure and needs to be described as a bounding object, e.g. a box or a circle, relative to which objects in the complex are positioned. Finally, a compartment, which typically corresponds to a cellular structure in a biological system, consists of a bounding object, e.g. a box or an oval. The objects inside are positioned using the respective bounding object as a reference frame. Species inside a compartment are not treated implicitly as interacting, as in the case of a complex. Nevertheless, they can be connected by reactions and their relative position to the compartment needs to be considered, e.g. to indicate luminal or transmembrane proteins.

Biological reactions require a more complex language than edges in regular graphs. The complexity of the encoding is affected by two main factors: the hypergraph nature of the molecular networks and the need to encode reaction types. Firstly, a reaction typically connects more than two species, e.g. multiple reactants, modifiers and products; hence, the considered networks are hypergraphs with reactions being hyperedges. Secondly, the role of a species in a reaction may affect its layout and the data format needs to be able to take this into account. Thirdly, one needs to be able to encode reaction type to visually distinguish relationships between the reactants and products, e.g. state transition, transport, as well as the role of the reaction modifiers, e.g. catalysis, inhibition. Both factors are necessary to consider, for instance, when fine-grained semantics of a reaction are captured via Boolean relations between conditions, under which a reaction can proceed.

### Data formats

Although all layout-aware exchange formats encode the intricacies of the systems biology diagrams as described above, they have their limitations and specifics. In the following paragraphs we briefly review the main formats focusing on the visual encoding parts of the languages and on the differences between them. A concise overview of the differences between the discussed formats can be found in Table 1.

**Table 1:** Support of the main layout and render-relevant features for species, complex species, reactions and compartments

	**SBML**	**SBML-CD**	**SBGN-ML**	**Remarks**
Set position	YES (L)	YES	YES	
Define shape	YES (R)	NO	NO	SBML-CD and SBGN-ML allow one to specify size of elements but not their shape.
Set color	YES (R)	YES	YES	
Set label position (L)	YES (L)	NO	YES	
Set label font (R)	YES (R)	YES (−)	YES	SBML-CD does not allow one to set font size for compartments and reactions.
Actively developed	YES	NO	YES	
Supports species modification	YES	YES	YES	
Supports node duplication	YES (L)	YES	NO	SBGN-ML only allows one to specify ‘clone’ flag for a glyph, thus the information about which species is actually cloned is missing.
Description of different biological granularities in the same diagram	YES	YES	NO	SBGN-ML does not allow one to mix the activity flow (AF), process description (PD) and entity relationship (ER) notations.
Multiple layouts	YES (L)	NO	NO	

If relevant, the SBML column includes information about which package supports given functionality (L = layout, R = render). Moreover, the multi package is required to support complexes and species modification definition in SBML. Please note that here we focus only on the major features and do not consider features such as ability to set border width or background opacity.

### SBML

SBML is the community-driven standard that was initially designed to encode molecular networks; however, nowadays it can be used to encode processes and models beyond systems biology such as population dynamics, etc. Besides its ability to encode molecular network information, SBML provides the ability to store visual diagrams via its layout and render packages. These extend the core language of SBML, used for describing the structure and dynamics of the underlying system.

SBML packages define XML schemas enriching the SBML core by adding XML tags that are placed at the root of the SBML document and thus do not interfere with core XML tags. The entities described in the core part of a document are then referenced from an extension using unique identifiers of species and reactions. In this way, it becomes easy to extend an existing document by package-specific nodes. SBML core tags are used to distinguish only basic types of entities such as species, reactions and compartments. To further distinguish types of species, one can assign a Systems Biology Ontology (SBO) term [17] to any element in the model. SBO terms add additional semantics to the model, which can be then utilized by software tools, e.g. to determine the shape of an element based on its type. Additional levels of semantics can be obtained by using annotations. SBML annotations have a standard format recommended by SBML specification. Using this format, entities can refer to controlled vocabulary terms and database identifiers that define and describe biological and biochemical entities.

The layout package [11] uses the notion of glyphs to encode information about positions of species, compartments and reactions. Species (including complexes), reaction and compartment glyphs use references to map to core elements. Species and compartments glyphs are described by a position and dimensions, i.e. by their bounding boxes. Reaction glyphs consist of lists of curved segments and species definitions involved in a given reaction. Each of the segments includes its type (line, cubic Bezier) and position needed to specify a segment. The species information in a reaction glyph contains not only the identifier to match a given species with its definition in the core but also the role of the species. Although reaction information in the core also contains information about the roles of the species, its role in the reaction glyph is more detailed, distinguishing, for example, the main substrate from a side substrate or an activating modifier from an inhibiting modifier.

By separating a layout from its structure, the layout package can also handle duplicate nodes. Node duplication is a technique that helps to increase clarity of a diagram. Duplicating species that are highly connected or species far apart in the diagram allows one to reduce edge crossings, which is considered as one of the main aesthetics criteria in graph drawing [18]. This is especially true in networks where small molecules such as water are involved in many reactions. The layout package allows for specifying multiple species glyphs referencing a single species definition, thus duplicating that species in the diagram. Such ways of representing duplicates also retain connections between the duplicates and the original molecule.

The layout package also allows for storing multiple layouts for the same network, which can be useful in complex cases, e.g. networks combining signaling, gene regulatory and metabolic reactions. In such cases, different aspects of the underlying biological system can be highlighted by laying out the relevant processes in different ways.

The render package [12] is an extension of the layout package, which provides a way to encode precise rendering of the elements. The render information consists of a set of styles that can be assigned to objects in the layout package. It is thus possible to define a visual object, such as a polygon filled with a given color or pattern, and assign it to a class of objects, for example all elements in the product role. Although the render package allows one to specify custom shapes for the elements, it is also possible to use the SBO terms attributes to derive the elements shapes when the rendering information is not available.

### CellDesigner’s SBML extension

CellDesigner is a popular diagram editor for creating SBGN-like networks, introduced long before the layout package was officially introduced into the SBML standard. Therefore, CellDesigner introduced its own SBML namespace, specifying a format to store the layouts and extending the syntax to encode any functionality not covered by SBML at the time of creation of the extension.

CellDesigner extends the SBML core by special tags for nested complexes and compartments, and species modification states. These functionalities are currently available in the SBML multi package [19]. With respect to entities positions, CellDesigner uses the notion of species alias that plays a similar role as the species glyph in SBML layout package. Unlike SBML, where the layout and rendering information are separated, CellDesigner species aliases contain some information about the rendering. Specifically, species alias tags include attributes allowing to specify font, color or line width of the respective bounding boxes. As for the rendering information, CellDesigner format does not define the shape of the species or reaction symbols. Instead, each species and reaction stores additional information in the CellDesigner extension, such as type (e.g. receptor protein, transcription) or modification (e.g. phosphorylation, catalysis). The rendering of the shapes corresponding to these features is hardcoded in CellDesigner software. This differs from the more general approach of the SBML render package, where shapes can be explicitly defined. Obviously, SBML render package can be used to fully implement the palette of shapes used by CellDesigner.

Some of the layout information in CellDesigner is stored directly in the SBML core. For instance, in the case of complex, polyline reactions, CellDesigner stores information about the midpoints in extension tags directly within the SBML core annotation tag of the respective reaction. For this reason, the CellDesigner encoding does not support multiple layouts. Regardless of this limitation, the prevalence of CellDesigner makes it a well-accepted format, handled by a number of the existing systems biology visualization and editing tools.

### SBGN-ML

Since for a period of time SBML did not support layout and render information, there was a need for storing an SBGN-compliant layout in a non-proprietary format. In effect, SBGN-ML [14] was proposed as the XML exchange format for encoding SBGN-compliant diagrams.

SBGN itself is a visual language, which strives to be unambiguous and consistent by using concise and discriminable symbols to represent molecular entities and processes. It consists of three sublanguages providing separate notations for three orthogonal types of diagrams: Process Description, Entity Relationship and Activity Flow [13]. The three languages cannot be mixed. Therefore, pathways containing, e.g. state transition reaction type (Process Description) and positive influence reaction (Activity Flow), cannot be captured in a single SBGN-compliant diagram.

Irrespective of the diagram type, SBGN standardizes graphical notations for types of nodes and edges to be used in a molecular diagram, but is agnostic to their colors, patterns, shades, shapes and thickness of edges or fonts. SBGN itself does not specify the diagram layout, i.e. positions of the elements. However, to serialize a network into a file to be shared among different programs, all the information not covered by the SBGN standard needs to be encoded as well. This is the role of the SBGN-ML. Importantly, SBGN-ML specification states that an SBGN-compliant diagram cannot mix the separate notations, which is a challenge, especially when representing heterogeneous networks.

SBGN-ML is a layout-focused format that does not separate the structure of the network from its layout and rendering information, contrary to SBML or CellDesigner. It uses the notions of glyphs and arcs to define both the network layout and its structure, and the layout is stored directly with the glyphs and arcs. Each glyph and arc contains information about the class (type) of a species or a process it represents. The list of available classes is limited by the SBGN sublanguage, in which a given network is described. Based on the class of an element or an interaction, the SBGN standard specifies how the entity should be rendered. Rendering information that is not specified by the SBGN standard (colors, font, width of edges, fonts) is then stored via extension tags that can be assigned to any SBGN element. The structure of such a tag should be specified via an XML namespace, which can link, for example, to the SBML render package [20]. Extensions are also used by SBGN-ML supporting tools to store other information, such as element annotations. However, in order for the extensions to be as general as possible, SBGN-ML does not define their structure. This, in turn, reduces the capability for sharing information among different SBGN-ML tools due to the lack of standardization.

Node duplication is an aspect which SBGN-ML lacks in comparison with both SBML and CellDesigner SBML, and which is difficult to solve via SBGN-ML extensions. Although SBGN provides visual specification for duplicated nodes, they are not related on the data level, and one can link duplicated species only by comparing their names and types. This can cause issues when manually editing such a species, because all the linked duplicates will need to be manually edited as well.

Finally, similarly to CellDesigner, SBGN-ML lacks the support for multiple layouts, which is understandable since SBGN-ML encodes the layout and underlying structure of the network by blending these two information layers together.

### Other layout-supporting formats

Although the three aforementioned formats are the most commonly used, there are other layout-aware formats. Two important examples are KEGG Markup Language (KGML) used in KEGG [21] and Graphical Pathway Markup Language (GPML) [22] used by WikiPathways [23]. However, these are tool-specific formats, used to support storage of networks within their respective platforms.

**Figure 1:**
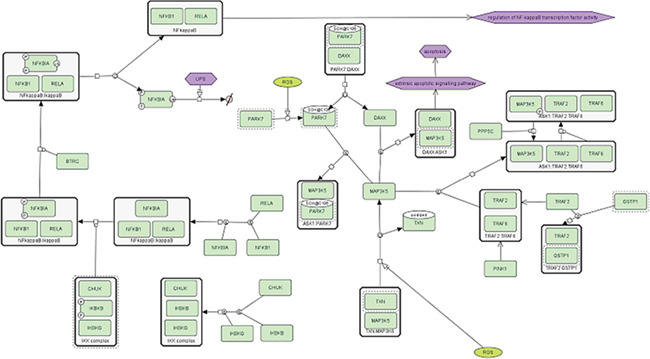
Inflammation signaling in neurons.

**Figure 2:**
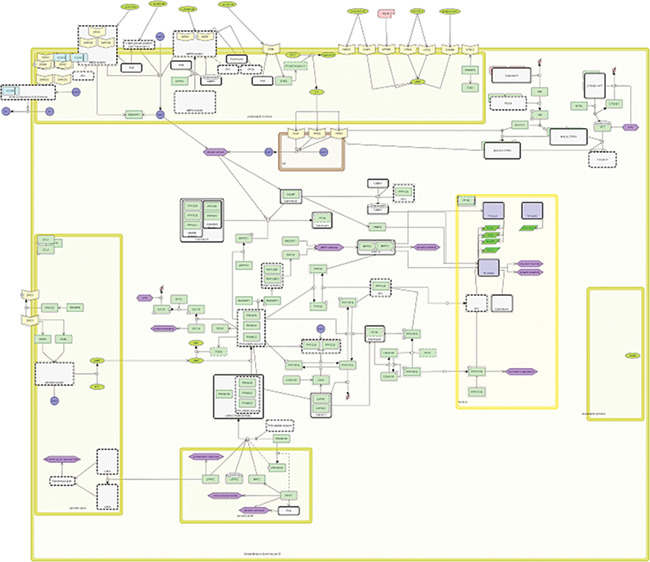
PD-related processes in SNs.

**Figure 3:**
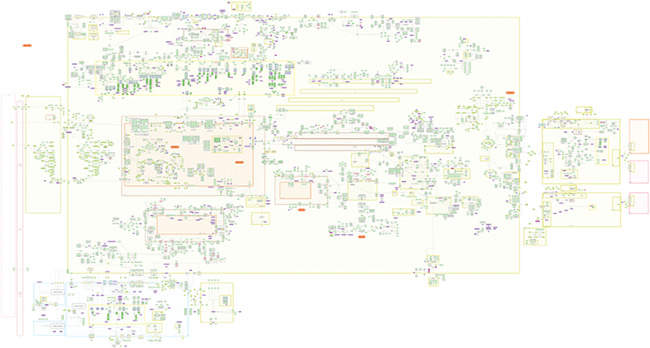
PD map, Spring ’18 edition.

### Support of layout in existing tools and repositories

Adoption of the layout-aware formats varies considerably between systems biology tools that deal with molecular networks. These tools range from those that do not support visualization at all (e.g. COBRApy [24], PySCeS [25]), through tools that support visualization, but do not support exchange of the layout information in a standard format (e.g. COPASI [26] or iPath [27]), to software platforms that directly focus on molecular network visualization and support exchange of the layout information using standard exchange formats (see below). In this section, we benchmark selected software solutions supporting layout-aware formats in systems biology using a set of three diagrams. We focus on the capabilities of available tools, in particular diagram editors, libraries and management platforms, to import and export diagrams in the three formats reviewed before: SBML, CellDesigner SBML and SBGN-ML.

#### Benchmark dataset

To test the support of the formats in existing layout-supporting tools and platforms, we prepared three layout-containing networks with increasing levels of complexity. Specifically, we used the Parkinson’s disease map (PD map) [28] from which we obtained three networks of different sizes: (1) the inflammation signaling pathway (INP) from the neuron compartment with 37 species and 20 reactions (Figure 1), (2) a multi-compartment network describing PD-related processes in striatal neurons (SNs) with 152 species and 132 reactions (Figure 2) and (3) the whole Parkinson’s disease map (PDM) consisting of about 5500 species and almost 2500 reactions in multiple compartments (Figure 3). All the networks contained duplicated nodes, proteins with modified residues and species grouped in complexes. Each of the three networks was exported from the PD map as a CellDesigner file and adjusted in CellDesigner. Each of the files was then converted using the implemented converter module (see Conversion module) to SBML with layout and to a SBGN-ML Process Description diagram. All the source data and their description are available as Supplementary Information.

#### Comparison of the software tools

In our comparison, we focus on tools that can store and export network layout in one of the above discussed systems biology exchange formats. For this reason, some recognized software projects such as COPASI [26] or JWS online [29] are not included, because they provide network visualization via tool-specific formats.

Table 2 summarizes the state of support for the data formats in current tools, libraries and network management platforms. We focused on three groups of available software diagram editors, libraries and management platforms. In this way, we would like to consider separately three aspects of working with systems biology networks, namely their creation, standardization and management of created content.

#### Diagram editors

Out of the seven tested diagram editors (CellDesigner [15], Cytoscape [30] + cy3sbml [31], Cystoscape [30] + cySBGN [32], Newt [33], Krayon for SBGN [34], PathVisio [22], Vanted [35] + SBGN-ED [36], yEd [37] + ySBGN [38]), only CellDesigner and Newt worked without major issues with our test networks. Obviously, CellDesigner can be used to edit its own files and support export to SBGN-ML, limited to the Process Description notation of SBGN. It should be noted that CellDesigner, unlike SBGN-ML, offers a notation similar to Activity Flow (Reduced notation) and allows for mixing between different notations. Export to SBGN-ML therefore supports only Process Description elements, which is a reasonable choice since it captures biochemical process in greatest detail.

The SBGN-ML versions of our test networks were without any issues opened only by Newt, which is currently the most versatile editor of SBGN-compliant networks, and Krayon. The Krayon editor had problems with assigning correct rendering order for nested compartments in case of SN and PDM, but apart from that, the import process was without any issues. It is worth pointing out that Newt allows the user to import layouts from CellDesigner, while CellDesigner itself is not able to fully export to SBGN-ML (see above). In this particular case Newt allows for mixing Process Description and Activity Flow notations which, however, produces diagrams that are not SBGN-compliant.

Both CellDesigner and Newt had performance issues when working with the PDM network. This is understandable in the case of Newt, which is a web-based platform using in-browser rendering of vector graphics to visualize the networks, which limits the number of visual elements that can be efficiently handled by contemporary browsers. Although CellDesigner is not limited by the constraints of the web platform, the deterioration in its performance was substantial as well. We could not identify the reason of this behavior as CellDesigner is a closed-source project.

Neither CellDesigner nor Newt support export to SBML with layout, let alone render, information. Support of SBML with layout and render is poor in general and the only diagram editor supporting SBML with layout is Vanted [35]. Although Vanted handled the layout information correctly, it did not show compartments in the SN and PDM networks. Another issue with Vanted is that it does not consider the rendering information at all, which is to be expected given the render package was released only recently. Thus, in the resulting visualization every element is encoded as an ellipsis.

#### Management platforms

Management platforms for systems biology networks include both pathway databases and custom platforms for visualization of contextualized networks, like disease maps. In this category of software tools, only MINERVA [16] supports layout encoded in SBML, CellDesigner SBML or SBGN-ML. NaviCell [39] platform supports only import from CellDesigner. BioUML [40] supports CellDesigner SBML and is supposed to support SBGN-ML. However, during import none of the three tested networks was recognized as SBGN-ML and export of the networks to CellDesigner format resulted in an exception.

The support of layout-aware formats in network repositories differs as well. The biggest repositories supporting visualization of molecular networks include Reactome [8], Pathway Commons [9], KEGG Pathways [21], WikiPathways [23] and BiGG Models [41]. Both Reactome and Pathway Commons store the underlying network in BioPAX and layouts in SBGN-ML. Moreover, Reactome uses SBML for the underlying network. BiGG stores its models in SBML (or COBRA-specific [42] files) while layout for models for which a diagram exists is stored in custom JSON files. These can then be loaded by Escher [43], a layout editing tool tightly bound with the BiGG Models platform. KEGG and WikiPathways both go in the direction of supporting their own formats, KGML and GPML, respectively.

**Table 2:** Support of layout-containing formats in existing software tools

	**Software tool**	**SBML (with layout)**		**SBML-CD (with layout)**		**SBGN-ML**		**Remarks**
		**Import**	**Export**	**Import**	**Export**	**Import**	**Export**	
Diagram editors	CellDesigner v4.4. [15]	Yes (−)	Yes (−)	Yes	Yes	No	Yes (−)	Supports export and import only from SBML v2 (without layout)
								Export to SBML with layout should be possible via the CellDesigner plugin [41] available within the JSBML project. However, using the plugin with the latest release of CellDesigner (4.4) results in empty plugin menu. Another option is using the CellDesigner parser [42], which worked for the INP and SN networks, but resulted in an exception with `NumberFormatException’ for the PDM network.
								Supports only PD SBGN, thus some reaction types cannot be converted to SBGN-ML.
	cy3sbml [31]	No	No	No	No	No	Yes	Although SBML layout support declared, it was not able to load correctly positions in SBML layout; shapes not supported.
								It is able to load CellDesigner SBML but drops layout.
	cySBGN v1.2.0 [32]	No	No	No	No	–	Yes	Available only for old version of Cytoscape (2.8.3)
								Loading of all the tested networks failed with ClassCastException (provided test SBGN files could be loaded so the issue was not related to plugin installation)
	Newt v1.1.0 [33]	No	No	Yes (−)	Yes	Yes	Yes	When importing a CellDesigner file, positions of some complexes are not well preserved.
								Supports PD and AF SBGN diagrams; allows one to mix PD and AF resulting in SBGN non-compliant diagram.
	Krayon for SBGN v1.0.1 [34]	No	No	No	No	Yes (−)	Yes	Supports PD SBGN diagrams.
								Does not show bounding boxes for some of the compartments in SN (e.g. nucleus) and PDM (e.g. several microtubules) due to incorrect rendering order.
	Vanted [35] + SBGN-ED [36]	Yes (−)	–	No	No	Yes (−)	Yes	Can load SBML-CD, but without layout.
								Can load SBML with layout, but does not handle compartments and render information. SBML export fails with an error message.
								Not capable to import SBGN-ML exported from Newt.
	yEd [37] + ySBGN [38]	No	No	No	No	No	Yes	Generic graph drawing software supporting export to SBGN-ML via ySBGN plugin.
	PathVisio v3.3.0 [22]	No	No	No	No	Yes (−)	Yes	Should be able to load in SBML, but importing any of the test networks led to `NullPointerException’.
								Importing the INP network SBGN-ML results in `ConverterException’.
Libraries	libSBML [44], JSBML [45]	Yes	Yes	No	No	No	No	Both libSBML and jSBML support the SBML including its extensions.
	libSBGN [14]	No	No	No	No	Yes	Yes	
Management platforms	MINERVA [16]	Yes	Yes	Yes	Yes	Yes (−)	Yes (−)	Supports PD SBGN, thus some reaction types cannot be converted to SBGN-ML.
	NaviCell [39]	No	No	Yes	No	No	No	
	Bi [40]	No	No	Yes (−)	No	–	–	Imports SBML without layout. Positions in export to SBML are stored in annotation tag containing SBGN-ML of that network.
								Positions of some complexes and reactions of the PDM network in SBML-CD were not recognized.
								SBGN-ML support declared, but none of the SBGN-ML files was recognized during import. The export of loaded networks to SBGN-ML resulted in `null’ error.

The minus denotes issues with given functionality in at least one of the tested networks; gray areas correspond to failures of the respective program.

#### Libraries

For both SBML and SBGN-ML languages, libraries are provided, which support programmatic manipulation of the networks. LibSBGN [14], the SBGN-ML manipulation library, has support for Java and C++ languages, while libSBML [44], the main SBML manipulation library, supports C, C++, C#, Java, Python and Matlab languages. Alternatively, one can also use JSBML [45] to work with SBML files.

#### Conversion between formats

With different sets of formats supported by different tools and repositories, tools for (batch) conversion between formats emerged. Conversion between CellDesigner SBML and SGBN-ML is provided by the cd2sbgnml conversion tool [46]. The JSBML project contains a CellDesigner plugin [47] that should support export to SBML with layout. However, using the plugin (downloaded March 2019) with the latest 64-bit release of CellDesigner (4.4) resulted in an empty plugins menu and no error message. Furthermore, there exists a bidirectional converter between CellDesigner and SBML with layout [48] that worked well with our benchmarking networks with the exception of the PDM network (see Table 2). KEGG [49] can be converted to SBGN-ML with SBGN-ED [50] and to CellDesigner SBML with KEGGtranslator [49, 51]. EscherConverter [43] provides export of Escher JSON files to both SBML with layout and SBGN-ML. Another converter between the non-layout SBML and other systems biology formats is the Systems Biology Format Converter (SBFC) project (SFBC has SBML to SBGN-ML converter module that is ‘in progress’ as of writing this paper. Moreover, it seems not to take into account layout since all the bounding boxes in the resulting SBGN-ML have zero coordinates and dimensions) [52]. However, when the SBML layout is required, one has to settle with import and export possibilities of individual tools such as Vanted, which also has issues (see Table 1). Theoretically, one could also use the online SBML Layout Viewer [53] (last updated in 2011) to export SBML files with layout and render information to PNG, PDF, SVG or TEX files. However, at the time of writing of this paper, only export to PNG was functional. Also, the viewer did not seem to take the render information into account at all even for the simplest system of one reaction with two species (available in Supplementary Information). We are not aware of any other converter supporting SBML with the layout or render package. This situation obviously hinders adoption of SBML layout and render standards and reinforces the current status quo when layout of the network is stored alongside the network itself, which complicates management of networks and adoption of visualization in existing projects. For this reason, we established the MINERVA conversion module.

## Conversion module

Because the formats for storing layouts were developed largely side by side, they share many concepts. In consequence, the semantics of layout-aware languages are largely overlapping. Therefore, it is possible to convert between the formats, often without losing any information in the process. Transformation between CellDesigner’s SBML extension and SBML with layout and render packages is possible except for multiple layouts (SBML only) and ability to encode graphical elements that are not network elements (CellDesigner only). The situation is more complicated when converting from SBML with layout and render and CellDesigner’s SBML extension to SBGN-ML, because in SBGN-compliant diagrams elements from different SBGN sublanguages cannot mix. However, in many situations complicated systems are drawn into a single diagram, which then needs to represent knowledge of varying detail. In some cases, the available information about a process may be insufficient to use the Process Description language, but it may be enough to describe the process with the Activity Flow notation. The solution is either to support Process Description diagrams only and not to encode some of the processes or to mix incompatible notation in one diagram.

### Import and export capabilities of MINERVA

We addressed the issue of conversion between layout-aware formats in a recently developed tool, which allows users to convert between SBML with layout and render packages, CellDesigner SBML and SBGN-ML Process Description diagrams. The tool is implemented as a conversion module in the MINERVA platform [16] and is accessible via publicly available REST API.

MINERVA is a web-based platform for hosting molecular interaction networks providing their visualization, exploration, automatic annotation of species, overlay of high-throughput or genetic variation data or visualization of available structural information [54]. In order to host various types of interaction networks, MINERVA imports and handles a wide range of formats. Currently, the platform supports all three discussed notations: SBML, CellDesigner SBML and SBGN-ML. To be able to handle the specifics of individual formats, its internal data structures capture a superset of available notations. MINERVA also supports export of the whole networks or arbitrary subsets of them to SBML, CellDesigner SBML and SGBN-ML Process Diagrams. In the case of SBML, MINERVA fully supports the layout package. The render package is used to encode all visual characteristics of the network elements except shape, which is encoded using the SBO term assigned to the elements and visualized using the CellDesigner palette for species and reactions. Additionally, MINERVA provides export of networks into SVG, PDF and PNG graphical formats.

### Conversion module interface

In MINERVA, the user primarily uses the GUI to interact with the hosted networks and administer them. Therefore, to carry out the conversion using the GUI, one needs to go through a point-and-click-based conversion process. This is useful for manual processing, but cumbersome if batch conversion was required.

Since version 11, MINERVA supports access to the hosted networks through a REST API [55]. To be able to programmatically convert between MINERVA supported input and output formats, we linked the import and export functionalities of MINERVA and provided access to this implementation by extending the API by new calls: (1) an API call to query which input and output formats (systems biology or graphical) are currently available, (2) an API call to convert between formats and (3) an API call to export an input format to a graphical format. The conversion API endpoints require to pass the input network in the body of a request and returns the network in the specified output format, which can be either a text format or binary format (e.g. in case of PNG). Currently, (MINERVA version 12.1) the input formats include SBML, CellDesigner SBML and SBGN-ML. The output formats include SBML, CellDesigner SBML, SBGN-ML, PDF, PNG and SVG. The architecture of the conversion module links the API to all importers and exporters provided by the queried MINERVA instance. Thus, when a new importer or exporter becomes available in MINERVA, it will be automatically available through the REST API keeping it in sync with the core MINERVA functionality. The new API is fully described in the documentation [56], including example calls.

The conversion functionality available via a REST API is immediately available, and when the platform itself is updated, the conversion functionality will be updated as well. One only needs access to a MINERVA instance which supports this functionality. The API can then be accessed from any programming language or simply from the command line using the cURL tool (see examples in the documentation [56]).

## Discussion

The existence of three closely related layout-aware formats for exchanging molecular networks is the consequence of parallel efforts and reflects the importance of layout. To address the needs of the quickly evolving domain of systems biology for visualization of complex diagrams, two of these formats are refined by ongoing, community-based work, while CellDesigner is an effect of a decade of work of the Systems Biology Institute in Tokyo.

CellDesigner extension of SBML is a relatively broad format that is able to represent most of the aspects of heterogeneous molecular networks. Unlike SBML and its CellDesigner extension, SBGN-ML was not developed as an all-purpose molecular exchange language, but rather as a layout-oriented language. As the layout implicitly contains the structure of the underlying network, SBGN-ML can be also used as a network exchange format. However, it lacks many aspects of SBML such as support for encoding node duplication, annotations, kinetics or rendering. For this reason, it is commonly used in repositories to store only the layout information [8, 9], while the underlying network is stored in other formats such as BioPAX or SBML. This situation is problematic, because the situation, where the layout and the content of a network are stored in separate files, prevents existing tools that need both data sources from actually using layouts. For example, to be able to model the flow of metabolites in a network and to study the effects of various perturbations of the system, one needs to use the SBML format to access the reaction rates and parameters. Should the outcome of the modeling be visualized on a diagram, one also needs to access the layout information for the same network, stored in an SBGN-ML file. This raises multiple issues, one of them being the difficult-to-manage multiple files for a single network. Moreover, in order to visualize results of the modeling, molecular entities in the two network files need to be mapped onto each other. Since the SBML and SBGN-ML files do not share common identifiers, obtaining the mapping is highly prone to errors. This issue is further complicated in case of handling of duplicates, where one species in SBML can correspond to multiple species in SBGN-ML. This obviously can discourage many developers from implementing visualization of stored layout, even though such visualization can be beneficial for interpretation of results and ease of use of given software. One way around this issue could be using the recently proposed COMBINE archive and OMEX format [57]. A single OMEX file can store the network model together with associated information, including the layout. Although the OMEX format could simplify the situation with sharing multiple files for a single network, the issue with mapping of modifiers remains. Moreover, we are not aware of any major projects currently supporting this format.

Simultaneous support for the three abovementioned layout-aware formats is a challenging task. Among all of the available tools discussed in this article, the management platforms are the most well positioned to tackle this challenge, as they reflect the meeting point between drawing the systems biology diagrams and utilizing them for research. The MINERVA Platform, initially developed to handle CellDesigner files, currently supports the SBML layout, SBML render and SBGN Process Description, and SBGN Activity Flow is foreseen in the upcoming versions. A thorough comparison of the cross-conversion of the three formats shows similarities between the concepts of network models for complex biological processes. Harmonization and integration of these efforts will greatly increase the utility and coverage of all concerned repositories, including pathway databases, disease maps and reconstruction databases.

We strongly believe that the adoption of SBML with its new packages may be an answer to the current unsatisfactory situation. Being a community-accepted standard, SBML with the layout package encoding the positional information, the render package supporting SBGN-compliant rendering (or any other visual language specification, for that matter) and the multi package supporting the long missing proper support for complexes and species modification, has now the potential to become the convergence point in the development of standards for representation of systems biology diagrams. It should be emphasized that rendering information can easily be determined by visualization software from SBO terms assigned to entities. A community-approved mapping of SBO terms to SBGN notation of glyphs allows for SBGN-compliant visualization without the need for direct support of the SBML render package.

## Conclusion

The current situation, when one format is used for encoding the structure, dynamics and annotations of molecular network and the other is used representing layout, is clearly unsatisfactory. This is further complicated by the coexistence of multiple layout-aware formats. We showed that the difference between these formats is not substantial; however, the support for encoding complex networks when varying level of understanding to processes is still missing.

We believe that the recent development of SBML paves a way toward a unification format, where SBML may be used as a notation to encode network structure, dynamics and layout, together with SBGN as a standard to visually encode the appearance of entities and their relationships. This is especially promising, since tools supporting serialization to SBGN-ML could easily implement serialization to the layout and render package augmented SBML.

To at least partially address the current situation, we introduced a conversion module for the MINERVA platform that allows the user to transform different layout-aware formats. Having conversion functionality available through the REST API allows for easy batch processing. The API can also be used to implement a preview of networks in any online resource through its ability to export a network to a chosen graphical format.

**Key Points**

- With increasing sizes of molecular networks, storing layout information together with the underlying network structure becomes a necessity.
- There exist three main layout-aware data formats: (i) SBML with layout and render extensions, (ii) CellDesigner’s SBML extension and (iii) SBGN-ML.
- No single tool, management platform or library supports all three main data formats.
- A new conversion module within the MINERVA platform was introduced providing the ability to convert between layout-aware formats and to export layouts into several graphical formats.
- Analysis of the formats shows that SBML with its extensions can serve as a unification format to encode network structure, dynamics and layout, together with the appearance of entities and their relationships.

## Supplementary Material

supplementary_bbz067

## Notes

**David Hoksza** is a researcher at the Luxembourg Centre for Systems Biomedicine (LCSB) and Charles University, Prague, working on approaches for network and molecular visualization, data integration and structural bioinformatics.

**Piotr Gawron** is a researcher at the LCSB, developing tools for visualization and exploration of complex molecular networks.

**Marek Ostaszewski** is a scientist and a project manager at the LCSB, working on IT applied to knowledge management in systems biomedicine, in particular in Parkinson’s disease, including clinical research.

**Jan Hasenauer** leads research groups at the University of Bonn and the Helmholtz Center Munich. His research focuses on the development and application of methods for the data-driven modeling of biological processes.

**Reinhard Schneider** is the head of the Bioinformatics Core Facility at the LCSB. His team develops solutions for efficient data integration, interpretation and exchange between the experimental, theoretical and medical domains.
