# Supplementary material for: Closing the gap between formats for storing layout information in systems biology
Source: Brief Bioinform. 2019 Jul 5;21(4):1249–60. doi: 10.1093/bib/bbz067 (PMC7373180; doi:10.1093/bib/bbz067)
Supplement: supplementary_bbz067 [file supplementary_bbz067.pdf]

# Supplementary information: Conversion examples with MINERVA conversion API

david.hoksza@uni.lu

All the underlying data for this supplementary information are accessible at https://git-r3lab.uni.lu/david.hoksza/supplementary-layout-formats. Please also read section 1.2 if you want to reproduce the results.

## 1 Evaluation set description

To showcase how MINERVA can convert between CellDesigner SBML (SBML-CD), SBML and SBGN-ML, we utilized Parkinson's disease (PD) map (Fujita et al. 2014) (available online at https://pdmap.uni.lu) to extract three layout-containing networks with increasing level of complexity.

- Inflammation signalling pathway from the neuron compartment (INF)
  - 37 species and 20 reactions
- Multi compartment network describing PD-related processes in striatal neurons (SN)
  - 152 species and 132 reactions
- Full Parkinson's disease map (PDM)
  - about 5,500 species and almost 2500 reactions

All the networks contained colors, duplicated nodes, proteins with modified residues and species grouped in complexes, which made them difficult to convert because of different level of support of these features in the three tested formats.

Each of the networks was exported from the Parkinson's disease map using MINERVA's export functionality available via the context menu. For the INF and SN, the corresponding regions of the map were selected via *Select mode* → *Export as map* → *CellDesigner SBML*. The INF and SN files were then adjusted in CellDesigner. Specifically we:

- removed the "Layout annotation" layer. CellDesigner supports layers, visibility of which can be turned on or off in CellDesigner, and MINERVA uses the layers to export pathways layout information (pathways can cross multiple compartments). In order to get rid of information about pathways crossing the exported regions of the map (which are exported together with the required part of the map) and keeping only the compartment information, we removed the "Layout annotation" layer completely for each of the networks.

- adjusted the size of the layout to fit the sizes of the exported networks. This was needed because when exporting a region in MINERVA the exported network keeps the original coordinates of the full map resulting in a lot of free space especially if only a small region of a large map is being exported.

The resulting files are attached to this supplementary information.

## 1.1 Conversion process

In order to test validity of the conversion process and to see which features of the networks can be retained and which get lost, we took each of the three networks in SBML-CD and converted it to each of the two remaining formats (SBML and SBGN-ML) and back. In each step we used MINERVA API, first to do the conversion, but also to export the converted files into all three MINERVA-supported graphical formats, i.e., PDF, PNG and SVG. We chose to start the process with CellDesigner SBML, because, with respect to layout and render capabilities, it enables to store super-set of features compared to the remaining two formats.

## 1.2 Comment on reproducibility

April'18 edition of Parkinson's disease map (accessible at https://webdav-r3lab.uni.lu/public/pdmap/Releases/) was used to create the INF, SN and PDM networks, which are included in the attached archive. Besides the source networks (in the CellDesigner SBML format), the archive also includes a shell script to carry out the conversions, and target layout files accompanied by image exports from those files. The shell script connects to an instance of MINERVA framework running at https://minerva-dev.lcsb.uni.lu to carry out the above introduced conversion process.

Please note that the following results were gnerated with MINERVA version 12.2 running at https://minerva-dev.lcsb.uni.lu. But since MINERVA is being upgraded regularly, it is possible that results obtained in the future might differ from the ones presented here.

There are two ways to exactly reproduce the results:

- Install the respective version of MINERVA available at

  https://git-r3lab.uni.lu/minerva/core/tree/fdeab47e6803031d363ce33254c8d3a56498f94f and run the script using that instance as the target one.

- Use the attached Docker compose script to create a Docker container to compose a Docker image of MINERVA version 12.2. (see README.md in the *docker* directory for details). Then you can access the API of the MINERVA instance running in the built Docker container via the port 8080.

## 1.3 Results

### 1.3.1 SBML-CD → SBML → SBML-CD

| | SBML-CD | SBML | SBML-CD |
|---|---|---|---|
| INF | 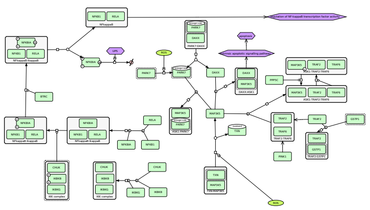 | 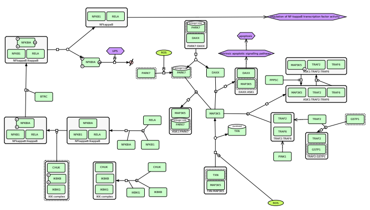 | 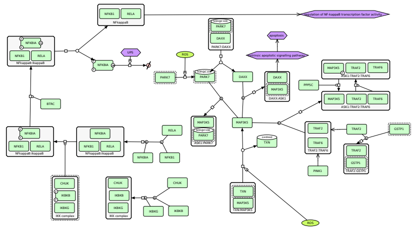 |
| SN | 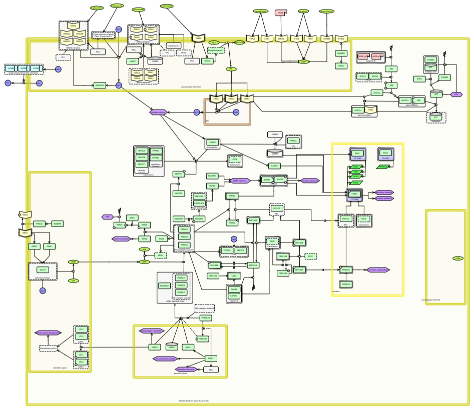 | 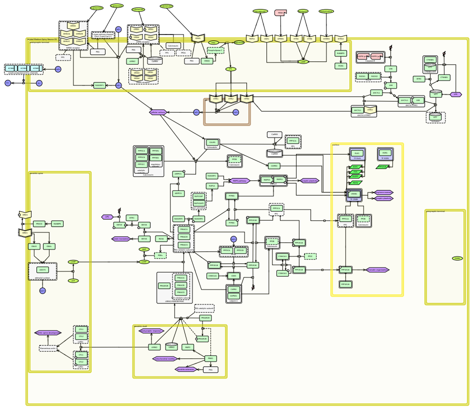 | 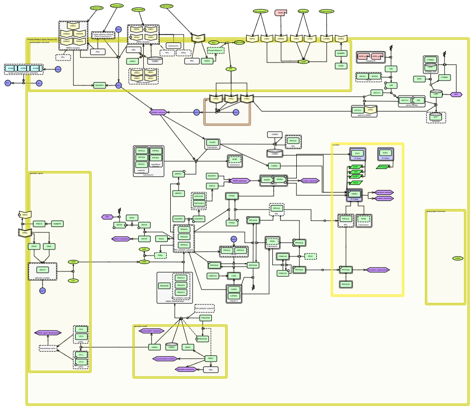 |
| PDM | 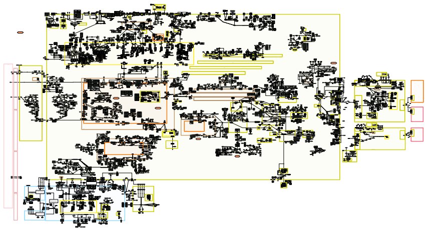 | 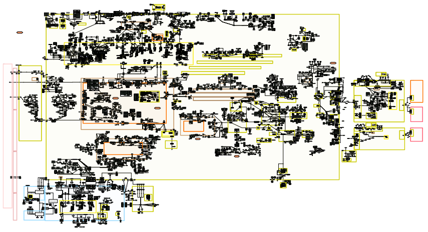 | 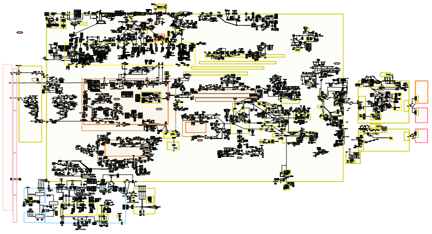 |

### 1.3.2 SBML-CD → SBGN-ML → SBML-CD

| | SBML-CD | SBGN-ML | SBML-CD |
|---|---|---|---|
| INF | 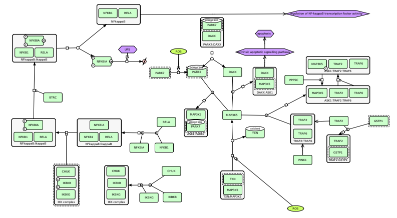 | 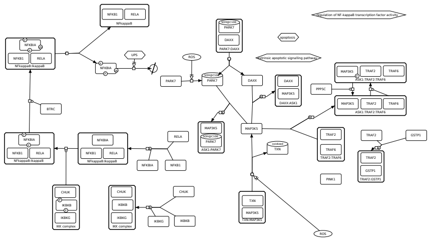 | 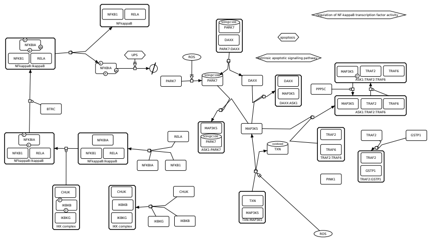 |
| SN | 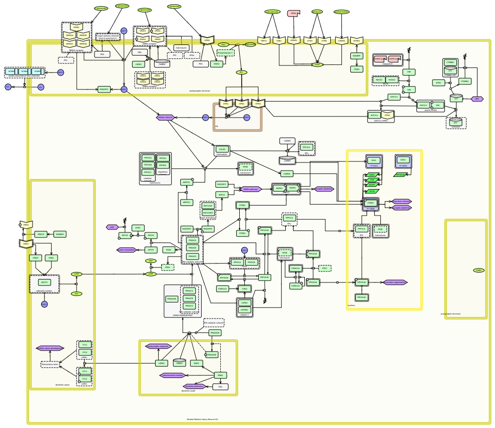 | 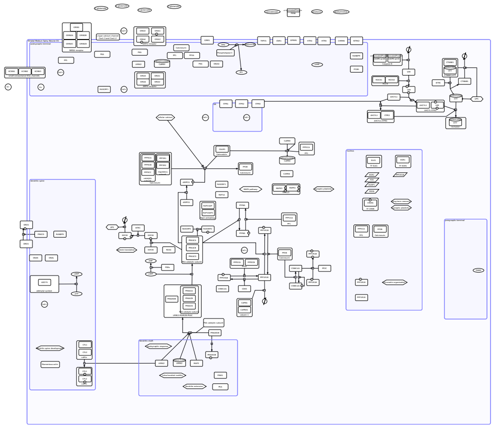 | 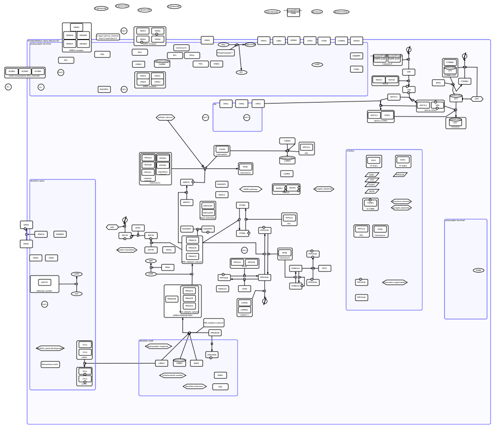 |
| PDM | 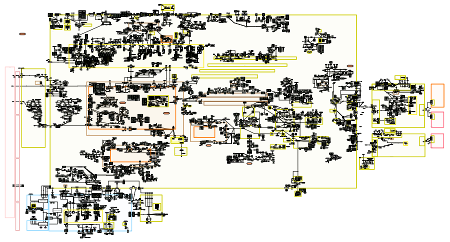 | 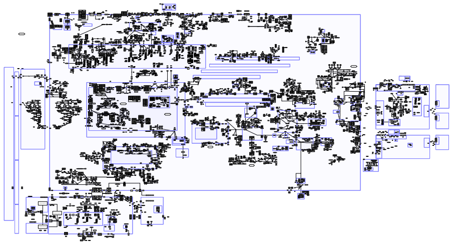 | 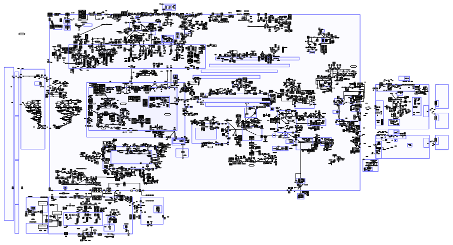 |

## 2 Discussion

### 2.1 SBML

The conversion from SBML-CD to SBML retains all shapes, colors, positions and compartments. The labels texts are preserved, but in case of compartments their position differs in the SBML version in all the networks. However, this does not impact perception of the networks.

Conversion from SBML back to SBML-CD retains all content fully.

### 2.2 SBGN-ML

The biggest issue when converting from SBML to SBGN-ML is that some reactions get lost because the starting networks contain processes of different levels of detail and therefore one would need to mix SBGN PD and AF languages to express the networks in SBGN-ML. This would, however, result in an invalid SBGN-ML document (see the main paper for discussion of this issue). Moreover, colors are not preserved, because SBGN-ML natively does not support any rendering information. Positions and shapes of entities are retained correctly.

Conversion from SBGN-ML back to SBML-CD retains all content fully.

## References

Fujita, Kazuhiro A. et al. (2014). "Integrating Pathways of Parkinson's Disease in a Molecular Interaction Map". In: *Molecular Neurobiology* 49.1, pp. 88–102. ISSN: 0893-7648. DOI: 10.1007/s12035-013-8489-4. URL: http://link.springer.com/10.1007/s12035-013-8489-4.
